# Management of children with poor prognosis first permanent molars: an interdisciplinary approach is the key

**DOI:** 10.1038/s41415-023-5816-7

**Published:** 2023-05-26

**Authors:** Shrita Lakhani, Fiona Noble, Helen Rodd, Martyn T. Cobourne

**Affiliations:** 598532468673084721434 Unit of Oral Health, Dentistry and Society, School of Clinical Dentistry, University of Sheffield, Sheffield, United Kingdom; 570317268767802090335 Centre for Craniofacial & Regenerative Biology, Department of Orthodontics, Faculty of Dental, Oral & Craniofacial Sciences, King´s College London, London, United Kingdom

## Abstract

Although there have been continuous improvements in child oral health over recent decades, first permanent molars (FPMs) remain susceptible to early caries and can often be affected by hypomineralisation. We highlight current thinking in caries management and the restoration of hypomineralised FPMs, while also discussing enforced loss of these teeth within the context of interceptive extractions or extractions as part of orthodontic treatment. Compromised FPMs can negatively impact on quality of life for a child and present significant management challenges for the dental team. Although a high-quality evidence base is lacking for the different treatment options, early diagnosis and multidisciplinary treatment planning are key to achieving the best outcomes.

## Key points

- Compromised first permanent molars continue to be a common clinical finding among children in the UK.
- Treatment planning should involve a team approach and rely upon careful assimilation of social, behavioural, medical and dental factors, alongside relevant preferences of the child and their family.
- Caries management and the restoration of hypomineralised first permanent molars is discussed, along with the consequences of enforced extraction of these teeth for the underlying occlusion.

## Introduction

Dental care for any child, especially those with high caries risk, should be founded on personalised and evidence-based prevention, aimed at averting disease and a host of potential negative impacts for the child, their family and service providers. A sizeable body of evidence supports the effectiveness of various professionally applied and home-care preventive regimens to help reduce caries and improve oral health outcomes for children.^1^^,^^2^

Given this preventive-based ethos, one may ask why dental health professionals are still seeing so many children with compromised first permanent molars (FPMs) in their daily practice. Moreover, how should these children be managed, given the complexity of decision-making in relation to long-term prognosis, orthodontic status, and relevant child/parental factors? Here, we provide a pragmatic commentary on the broad principles of dental care for children with FPMs of poor prognosis.

## Why are we still seeing children with compromised FPMs?

General dental practitioners (GDPs) in the UK have recently reported that around 10% of the children that they see will have compromised FPMs.^3^ Data from the Office of National Statistics (2015) corroborate this clinical impression, with the finding that 5% of eight-year-olds, and an alarming 25% of 15-year-olds, have some form of caries in their FPMs.^4^ It is also important to recognise that carious FPMs may have an underlying enamel defect, which can predispose them to a greater risk of caries. Molar incisor hypomineralisation (MIH) is an increasingly common systemic condition, characterised by qualitative enamel defects predominating in the FPMs and incisor teeth.^5^ Not only are affected molars more likely to develop caries (reportedly up to six times), but they are also prone to rapid and extensive post-eruptive enamel breakdown, which can cause extreme dentine hypersensitivity.^5^^,^^6^ Epidemiological data suggest that MIH affects around 13% of children worldwide, so even in communities with decreasing caries rates, clinicians will continue to face the challenge of managing children with poor prognosis FPMs.^7^

## Clinical management of children with one or more compromised FPMs

Treatment planning for children with carious and/or hypomineralised FPMs relies on the assimilation of social, behavioural, medical and dental factors, alongside child and family preferences. The European Academy of Paediatric Dentistry has recently published *Best clinical practice guidance*, specific to children with MIH, which provides a consensus for treatment alongside the quality of the supporting evidence for each option.^8^ It is interesting to reflect on reported differences in management approaches between various clinician groups and between different countries.^3^ Notwithstanding these acknowledged disparities, an early diagnosis of enamel hypomineralisation and/or caries is paramount to inform pre-emptive (simple) treatment and maximising best clinical and patient-reported outcomes over the longer-term.

### The initial assessment

It is important to carry out a comprehensive and timely history and examination for any child with FPMs of concern. As will be discussed later, the stage of dental development is an important factor when planning the timing of any extractions. Furthermore, clinicians should be aware of the potential for congenitally missing second premolars in children with MIH which would represent a contraindication to FPM extraction.^9^ Table 1 highlights some of the factors that should be elicited and taken into consideration for all children with compromised FPMs.

**Table 1:** Key points to include in a history and examination of children with FPMs of concern

History and examination	Further details
History of presenting complaint	Any acute pain or infection requiring immediate intervention? Nature and impact of any hypersensitivity from hypomineralised teeth?
Medical	Any medical condition placing the child at significant risk from a dental infection or treatment, including general anaesthetic (for example, immunocompromised, congenital cardiac condition, oncology)?
Past dental experience	Is the child a regular attender? Do they have experience of previous restorative or surgical treatment including use of local anaesthetic?
Oral hygiene and dietary practices	Is the child brushing twice daily with optimal fluoridated toothpaste? What are the children drinking and snacking on between meals?
Social	Are there any safeguarding concerns or family difficulties with attendance for multiple visits? What are the child's and family's expectations and wishes for treatment?
Behavioural	Is the child dentally anxious and/or potentially pre-cooperative with the proposed treatment?
Clinical examination	Note overall caries risk status (including carious or hypomineralised primary molars) Undertake a basic periodontal examination (for children >7 years) Carry out detailed assessment of FPMs in terms of extent/site of hypomineralisation, and any post-eruptive breakdown, consider if they are restorable Carry out a basic orthodontic assessment, note incisor and molar relationships and confirm that maxillary canines are buccally placed Check for any anomalies, such as microdont teeth, infraoccluded primary molars, missing teeth
Radiographic examination, including a panoramic radiograph and intraoral bitewings	Check stage of dental development (note the developmental stage of second permanent molars) Confirm all permanent teeth are present and check for developing wisdom teeth Determine caries extent and proximity to pulp (in all teeth, including FPMs) Check for any periapical pathology Check for any other dental anomalies

### Caries prevention and management of dentine hypersensitivity

**Fig. 1:**
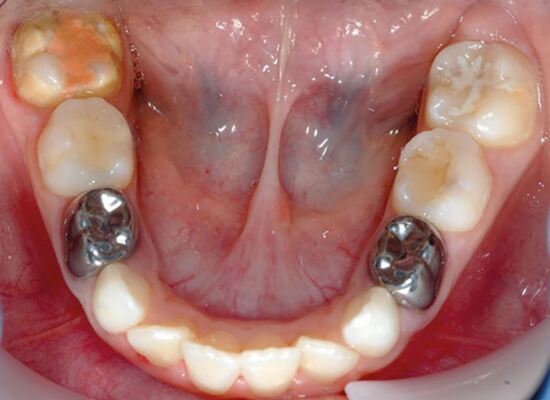
A seven-year-old child with high caries risk and hypomineralised lower right FPM. A conventional fissure sealant was possible on the lower left FPM but due to dentine hypersensitivity, a resin-modified glass ionomer cement was preferable on the lower right FPM

Having addressed any acute presenting complaint, the first phase of any treatment plan is to establish a preventive programme.^2^ Children with carious and/or hypomineralised FPMs require optimal topical fluoride regimens, including professionally applied fluoride varnish at least twice a year, 2800 ppm fluoride toothpaste (if older than ten years old), and, ideally, a daily fluoridated mouthwash, in conjunction with dietary advice and toothbrushing instruction. Fissure sealants should be applied on any permanent molars not requiring restoration or extraction, although bonding to hypomineralised enamel can be unpredictable.^10^ This, together with poor moisture control (stemming from an underlying dentine hypersensitivity and/or child anxiety) can lead to higher failure rates of conventional resin-based fissure sealants.^8^ An alternative and less technique-sensitive approach for both child and clinician is the interim use of a resin-modified glass ionomer sealant restoration (Fig. 1).^8^ Although some clinicians advocate the use of remineralising products (casein phosphopeptide-amorphous calcium phosphate products), desensitising toothpastes, or silver fluoride preparations for the management of MIH hypersensitivity, the evidence base has not been established.^10^ Having carried out an initial clinical and radiographic assessment (ideally soon after eruption of the FPMs) the initial phase of treatment aims to manage any symptoms or anxiety, establish a personalised preventive strategy, and protect the teeth from any further post-eruptive breakdown, caries or erosion. The next consideration is to evaluate the likely long-term prognosis and treatment need for each FPM, alongside the variables outlined in Table 1. However, a definitive decision may not be appropriate at the first assessment, so the child should be kept under regular review and the family made aware that there are several future treatment options.

### Taking a restorative approach

Current thinking regarding dentine or cavitated caries management is orientated towards minimally invasive approaches, which favour selective or stepwise caries removal, rather than complete.^11^ However, in the case of deep caries in asymptomatic vital FPMs, partial or coronal pulpotomies (using materials such as mineral trioxide aggregate or biodentine) have been reported to have variable success rates of around 60-80% at five years.^12^^,^^13^ Crucial to the success of these techniques is optimal moisture control with rubber dam and the use of restorative materials that provide an hermetic seal. The use of amalgam is no longer supported for children under the age of 15 in the UK.^14^ It is not common practice to embark on a pulpectomy for FPMs for this young age group in the UK and while endodontic treatment is possible (and sometimes indicated), extraction of these teeth (with or without orthodontic space closure) is likely to achieve better patient and cost outcomes in the longer-term.^15^

**Fig. 2:**
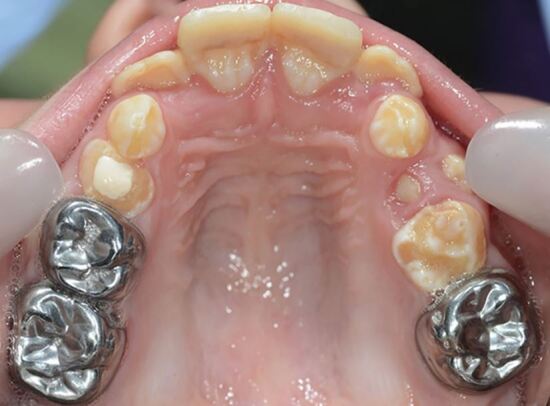
A nine-year-old child with upper anterior crowding, hypomineralised second primary molars and FPMs. Preformed metal crowns were placed (using the non-invasive Hall technique) as a mid-term restoration until the eruption of the second permanent molars and planned orthodontic extraction of the compromised FPMs

More challenging than simple caries management is the restoration of hypomineralised FPMs. A recent systematic review provides a comprehensive critique of the various restorative options for children with MIH.^10^ For mildly affected FPMs (minimal post-eruptive breakdown), a composite resin restoration, extending beyond the visibly affected enamel opacity, would seem to be the best option. In cases where the opacities involve multiple surfaces, together with rapid post-eruptive breakdown and hypersensitivity, direct or indirect composite resin restorations may be considered. Expert opinion seems to support the removal of any soft hypomineralised enamel before the placement of an indirect restoration with optimal rubber dam moisture control.^10^ For some children with severe MIH, full coronal coverage using a preformed metal crown (PMC) may offer a simple medium-term restoration. In such cases, the non-invasive Hall technique for PMC placement usually obviates the need for local anaesthetic and tooth tissue removal; beneficial for young and/or anxious children. A PMC is not considered a definitive restoration (due to potential wear and periodontal damage), but it can be advantageous in situations where the FPM needs to be retained for several years until the optimal time for its planned removal (Fig. 2).

Any restorative intervention for a young child with compromised FPMs will confer a long-term treatment burden for that patient. Indeed, between the age of 9-18-years old, children with MIH can undergo four times as many treatment episodes (usually retreatment of failed restorations) for these teeth compared to a control group.^16^ By the age of 18 years, the MIH group continued to face an ongoing cycle of restorative interventions.

### Indications for extraction

The removal of one or more compromised FPMs is not common practice outside the UK, and a restorative approach is generally favoured in Europe.^8^ This may be partly explained by the higher caries prevalence in the UK and more widespread societal acceptance of extractions under sedation or general anaesthetic. Nonetheless, it is argued that extractions may offer the most appropriate treatment for some children with extensive caries and/or hypomineralisation, particularly those experiencing symptoms.^17^ The rationale for FPM 'interceptive' extraction is that it obviates the need for ongoing restorative and endodontic care, and encourages second permanent molar eruption with space closure between this tooth and the second premolar, particularly if undertaken at the 'ideal' stage of dental development (that is, around the age of 8-10 years, with the second permanent molar still developing within alveolar bone). Variable success rates have been reported in the maxillary and mandibular arches, with most researchers citing an 80-90% chance of contact in the maxillary arch and around 50-60% in the mandible.^18^^,^^19^^,^^20^ However, the evidence base for clinical and patient-based outcomes associated with FPMs remains surprisingly sparse, with a lack of randomised clinical trials (RCTs) (Box 1). In general, there are some acknowledged clinical and patient-related factors which tend to favour the extraction of one or more compromised FPMs (Table 2).

**Table 2:** Clinical and patient-related factors which tend to support the extraction of FPMs of concern

Clinical factors	Patient factors
Severely compromised FPM (for example, deep caries or restoration, pulpal or periapical pathology, extensive hypomineralisation involving multiple surfaces, with associated post-eruptive breakdown)	Symptomatic teeth (caries-related pulpal symptoms or hypersensitivity relating to enamel hypomineralisation)
	Patient at ideal stage of dental development (8-10 years)
All permanent successors present	High caries risk
Presence of developing third permanent molars	Irregular or symptomatic attendance
Requires orthodontic extractions (of otherwise healthy teeth)	Dental anxiety or behavioural needs precluding restorative management with local anaesthetic/sedation

Box 1 Why are there no randomised clinical trials investigating the consequences of FPM extraction? Given the number of children seen every year in both the UK and internationally with compromised FPMs, it would seem surprising that there is a lack of high-quality data investigating the outcomes of treatment. Currently, much of what we know is based upon retrospective cohort data collected from busy hospital departments - particularly in relation to occlusal outcomes following enforced loss of these teeth. Not only do we need more robust data on the long-term occlusal and oral health-related sequelae of interceptive extractions or restorative care, but also more data relating to quality of life outcomes in relation to management decisions for children affected by this common condition over both the immediate and longer-term. It is generally acknowledged that a RCT represents the most robust method of investigating the effects of a treatment intervention, but there are significant challenges associated with applying this methodology to the management of compromised FPMs. Indeed, while attempts have been made to apply this methodology to FPM extraction, no results have yet been published.^21^ A significant issue is the ethics of randomising children to extractions or restoration, which is exacerbated by fundamentally different approaches to treatment in different parts of the world. Perhaps the solution might be an international prospective multicentre trial with good control of the variables involved, rather than the ethically more challenging imposition of a RCT.

### Orthodontic considerations

**Fig. 3:**
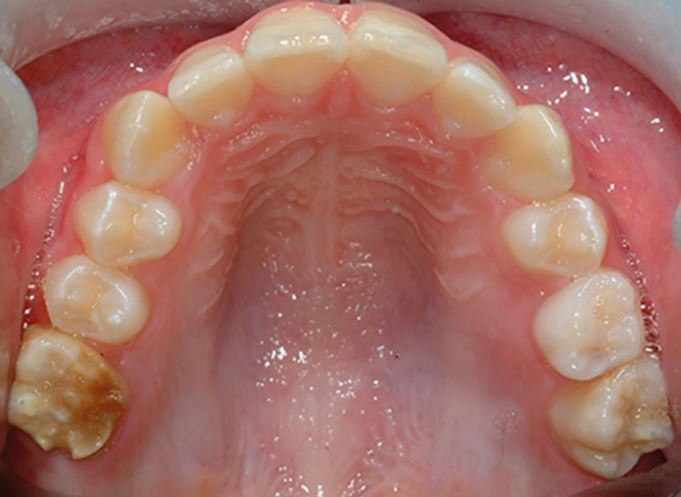
An 11-year-old child with poor prognosis hypomineralised upper right FPM showing brown opacities and extensive post-eruptive enamel breakdown. The upper left FPM has a small, cream-coloured occlusal opacity but no post-eruptive breakdown, and would be considered of good prognosis

The role of the orthodontist in managing poor prognosis FPMs is to liaise with the paediatric dentist or GDP, and give advice within the context of any potential interceptive extractions and overall management of any underlying malocclusion. It is important to state that the key to orthodontic decision-making is clear direction on the long-term prognosis of each affected tooth and this should come from the paediatric dentist or GDP, particularly in relation to teeth affected by MIH (Fig. 3). In addition, the presence of any acute symptoms, the ability of a child to accept restorative care, and of course, any requirement for a general anaesthetic as part of their management, will have a significant influence on fundamental treatment planning decisions.^17^ Guidelines from the Faculty of Dental Surgery, Royal College of Surgeons of England, describe best practice on the timing, compensation and balancing of FPM extractions; however, the evidence base is generally low quality, with a preponderance of retrospective investigations currently populating the literature.^22^

In terms of interceptive extractions, predictors for successful eruption of the second permanent molar have always been more important in the mandibular arch. Classically, the child should have a Class I malocclusion and be between the ages of 8-10 years old to ensure minimal disruption to occlusal development. In addition, radiographic evidence of the second permanent molar unerupted in alveolar bone and early mineralisation of the bifurcation represent an optimal time for FPM extraction to ensure a good eruptive position of the second molar.^23^^,^^24^ More recent evidence would suggest that the window of opportunity in relation to radiographic development of the second permanent molar is wider in terms of bifurcation mineralisation, and that the mesiodistal angulation of these teeth and presence of a third permanent molar can offer further useful prediction of favourable second permanent molar eruption.^19^^,^^20^^,^^25^ All these predictive factors are more relevant in the mandibular arch, as the maxillary second permanent molar will generally achieve a good eruptive position over a wider range of extraction timings.^18^^,^^19^^,^^20^ In terms of interceptive treatment, routine balancing extraction of a sound FPM to preserve a dental centreline is not recommended. Compensating extraction of a sound upper FPM has been suggested to prevent over-eruption of this tooth when extraction of the lower FPM is required. For an upper FPM that will remain unopposed for some time, significant over-eruption can cause interferences with the erupting lower second permanent molar, impeding space closure and potentially contributing to other occlusal interferences. Current evidence would suggest that the risk of upper FPM over-eruption, as a consequence of lower FPM extraction, is small, and decisions should be made on a case-by-case basis.^26^

**Fig. 4:**
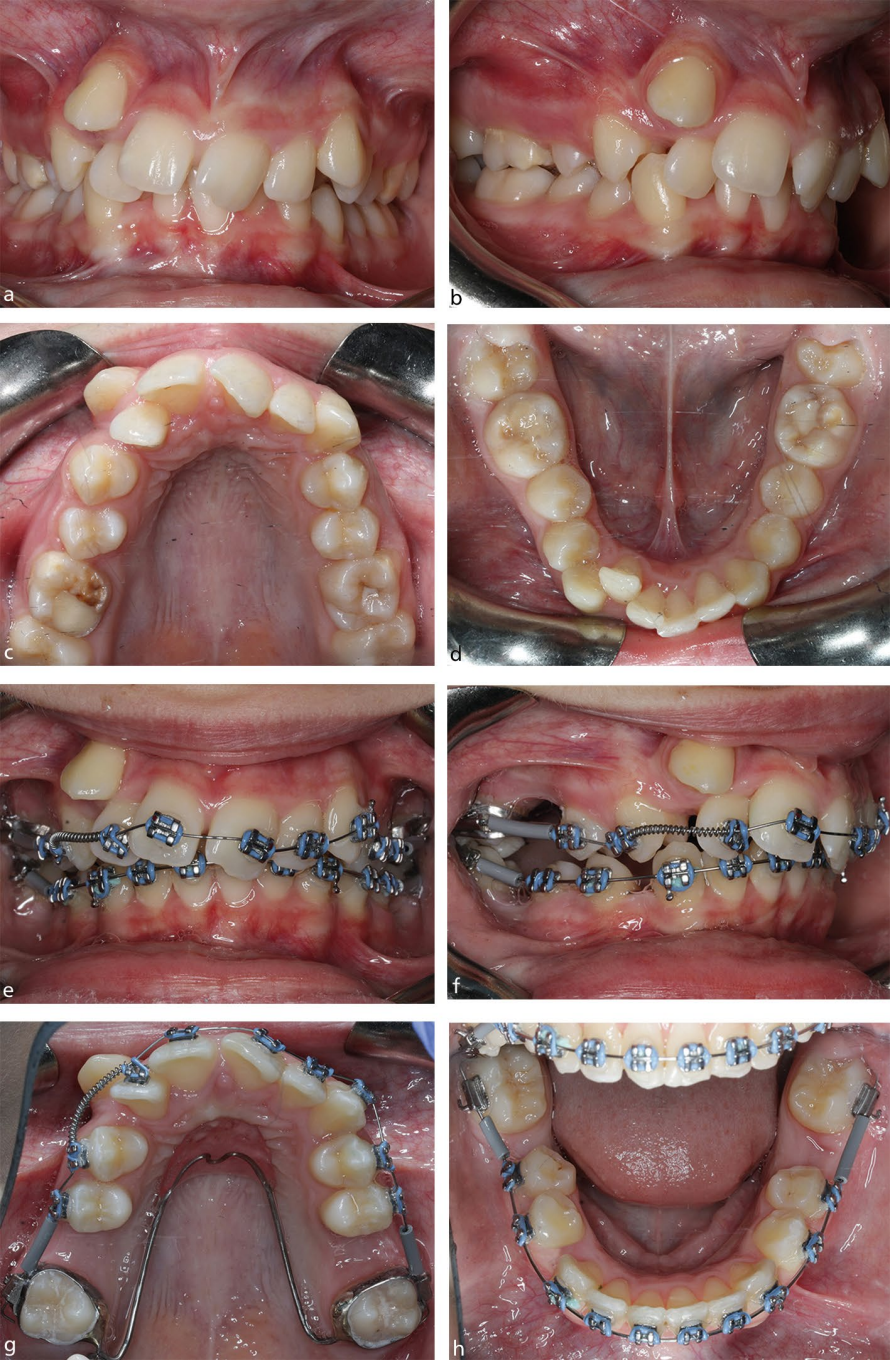
a, b, c, d, e, f, g, h) A 15-year-old child with a challenging Class II Division 2 malocclusion complicated by the presence of a significant sagittal discrepancy, severe crowding and compromised FPMs, being treated with fixed appliances. A transpalatal arch and Nance button have been placed but the anchorage demand remains high

**Fig. 5:**
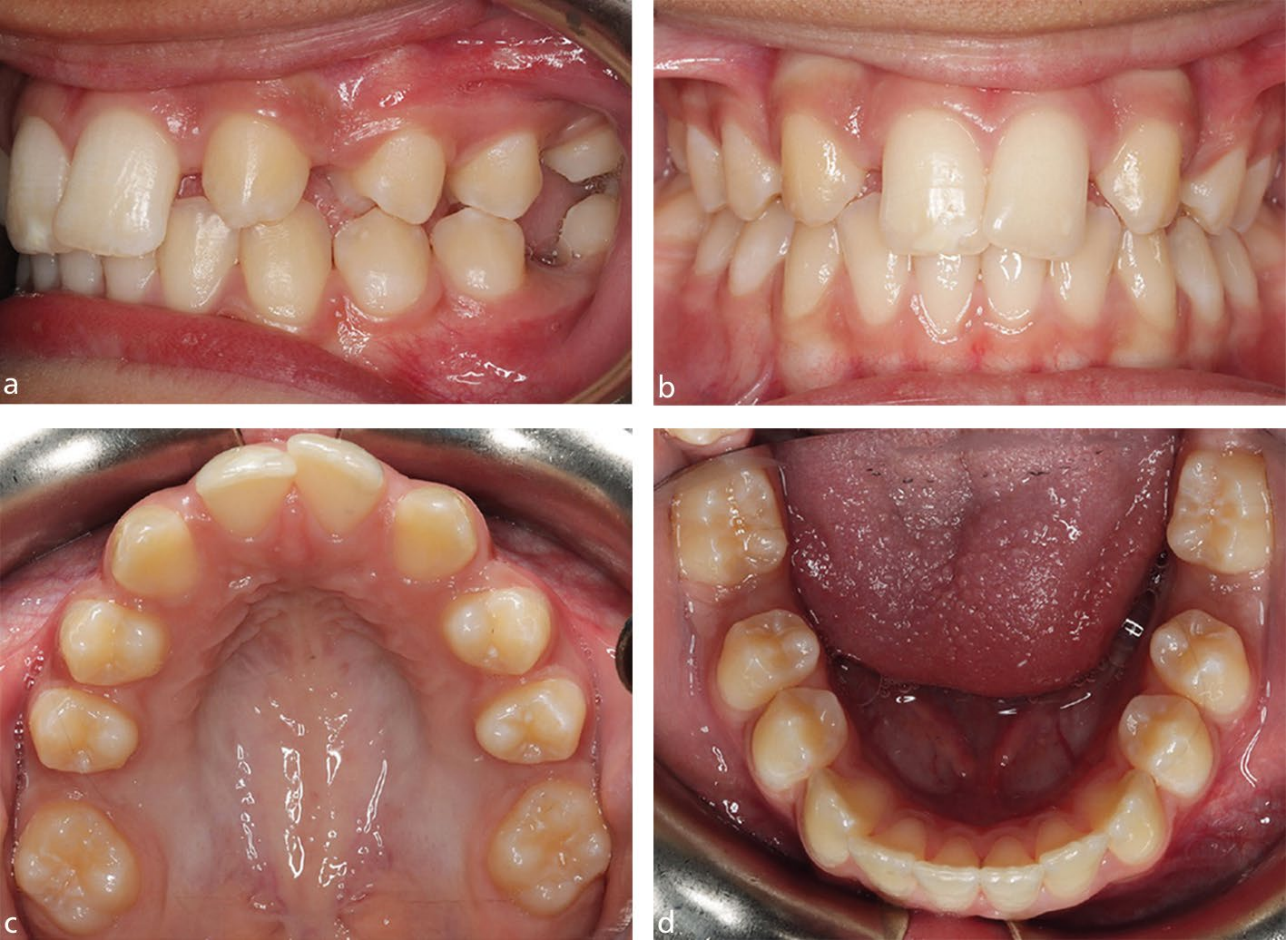
a, b, c, d) A 14-year-old Class I case with absent maxillary lateral incisors and previous interceptive extraction of all four FPMs. The eruptive position of the second permanent molars is poor in all four quadrants with generalised spacing present; however, the relatively mild nature of the malocclusion means that alignment and space closure is easily achievable with fixed appliance treatment

**Fig. 6:**
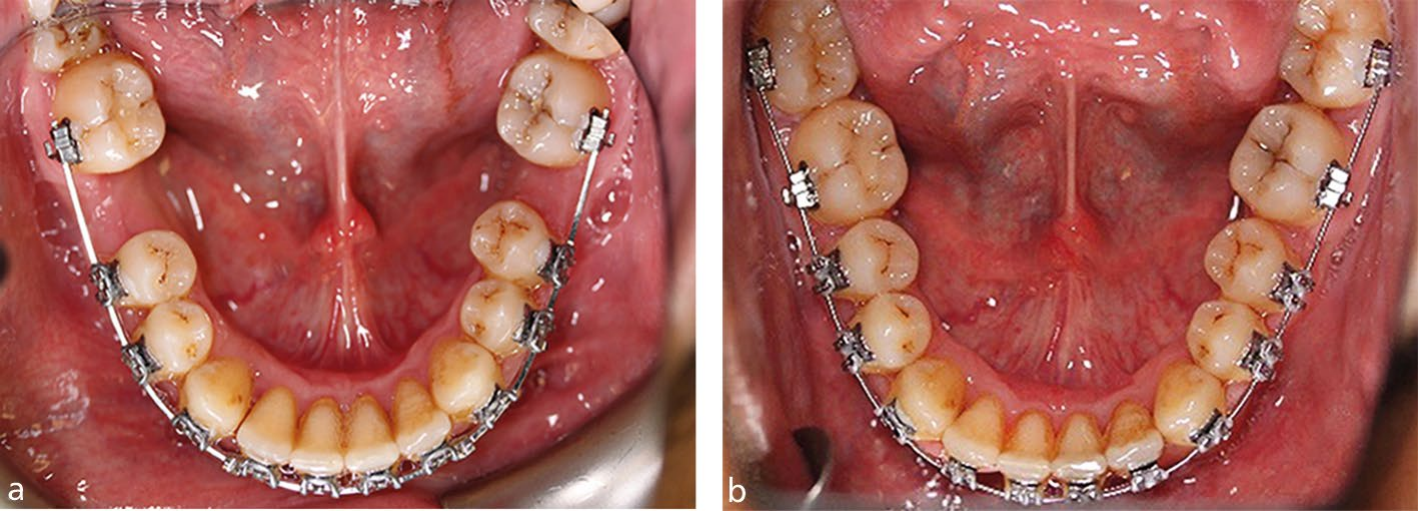
a, b) An adult Class I case requiring extraction of all four FPMs. Space closure and accommodation of the third permanent molars has been achieved with fixed appliances but this has taken over 12 months (and the lower second permanent molars will still require some minor modification of position)

The widespread use of modern fixed appliances and fixed anchorage in orthodontics has meant that the incorporation of FPM extractions has become more routine in the management of malocclusion.^27^^,^^28^ Indeed, with radiographic evidence of third permanent molar development and a requirement for extraction-based fixed appliance treatment, the presence of caries, MIH, or a restoration in any FPM should elicit serious consideration of its elective extraction as part of an orthodontic treatment plan incorporating fixed appliances. When considering orthodontic treatment, there is no doubt that occlusal outcomes are generally easier to control in Class I cases, and those cases associated with any degree of sagittal discrepancy that are at the milder end of the spectrum. In general, the higher the anchorage requirements, the more difficult FPM extraction cases become to manage with fixed appliances, particularly those associated with the presence of a significant overjet and/or crowding. The reliance upon anchorage reinforcement with headgear, transpalatal arches and mini implants becomes more important for achieving a successful outcome, particularly in the older child (Fig. 4). However, depending upon severity of the malocclusion, even poorly positioned second permanent molars can be relatively easily managed with fixed appliances (Fig. 5). Space closure can be prolonged, particularly in the mandibular arch, but careful anchorage management and patient mechanics can produce good occlusal results, even in the adult dentition (Fig. 6).

It should also be remembered that for some children presenting in the established permanent dentition with high caries risk and/or poor oral hygiene, fixed appliances might not be appropriate and sometimes compromises will need to be made when FPMs cannot be restored.

## Patient perspectives and oral quality of life

Within the dental literature, there is growing emphasis on how dental conditions may impact on children's oral and general health-related quality of life. It is now well-recognised that both untreated caries and MIH can have profoundly negative impacts on a child's social, emotional and functional wellbeing.^1^^,^^29^ More research is needed to better understand how interventions can improve patient-reported outcomes and experiences for children with compromised FPMs, both in the short- and long-term.^10^

## Conclusion

Compromised FPMs can have a negative impact on a child's quality of life and present significant management challenges for the dental team. Although a high-quality evidence base is still lacking to support all the different treatment options, early diagnosis and multidisciplinary treatment planning are key to achieving the best possible outcomes.
